# Micro-osteoperforations' impact on orthodontic tooth movement rate: Split mouth research

**DOI:** 10.6026/973206300222400

**Published:** 2026-04-30

**Authors:** Irfan Bashir, Syed Zameer Khurshaid, Nayeem Ul Haq Khanday, Syed Abid Hussain, G. Dinesh Kumar

**Affiliations:** 1 Department of Orthodontics and Dentofacial Orthopaedics, Government Dental College, Srinagar, Jammu & Kashmir, India

**Keywords:** Micro-osteoperforations, canine retraction, mini-implants, anchorage loss, visual analog scale

## Abstract

Micro-osteoperforations (MOP) accelerate tooth movement by enhancing osteoclastic activity thereby reducing overall treatment time. Therefore, it is of interest to report current research to evaluate the outcome of micro-osteoperforations on the rate of orthodontic canine retraction. We assessed their potential for accelerating tooth movement during fixed orthodontic treatment. Maxillary arch of 16 selected participants was randomly alienated into experimental and control side. After extraction of maxillary first premolars and completion of alignment and levelling, micro-osteoperforations were performed on the experimental side. 150-gram force was applied on both sides using NiTi closed coil spring for canine retraction. Tooth movement measurements were obtained at 4, 8, and 12 weeks to evaluate effectiveness of MOP's. MOPs effectively accelerate orthodontic tooth movement during the 1st 4 weeks of canine retraction. The technique does not increase tipping, anchorage loss, or patient discomfort.

## Background:

The emphasis on maintaining good health has increased the demand for orthodontic care. However, the prolonged duration of orthodontic treatment remains a primary deterrent for many patients particularly adults. It affects psychological wellbeing and increases the likelihood of periodontal problems, caries and root resorption [1, 2]. Consequently, finding strategies to shorten treatment duration without compromising results has become a key focus in orthodontic research. Current clinical approaches rely largely on mechanical forces to trigger bone remodelling and enable tooth movement. The rate of tooth movement depends on bone resorption, which itself is governed by osteoclast activity [3]. Thus, any factor enhancing the recruitment or differentiation of osteoclast precursors increases the rate of orthodontic tooth movement (OTM). Ongoing research therefore aims to increase the local concentration of these signaling molecules that stimulate this process. A relatively new technique known as micro-osteoperforations (MOP), involves creating small perforations on cortical bone with minimal discomfort. It is a minimally invasive procedure that facilitates faster tooth movement during retraction [4, 5]. The controlled micro-trauma created promotes the release of naturally occurring inflammatory mediators which subsequently accelerates osteoclastic activity and enhances OTM rate [1]. Therefore, it is of interest to evaluate the outcome of micro-osteoperforations on the rate of maxillary canine retraction.

## Materials and Methods:

The study commenced only after receiving approval from the Institutional Ethics Committee of Government Dental College, Srinagar, J & K (vide no. GDC/Ethic/10567/22, dated 05-09-2022). The research protocol followed the ethical standards set forth in the Declaration of Helsinki. Prior to enrolment, written informed consent was obtained from each participant. Participants were clearly informed about the nature and purpose of research, the procedures to be carried out and any possible risks and benefits. Patients were also informed about having freedom to withdraw at any stage without prejudice. Throughout the course of the research, strict measures were taken to ensure the confidentiality of participant's personal and medical information. Inclusion Criteria were; subjects aged 16 - 30 years , of both genders, with Average growth pattern, Class II Div1 malocclusion cases indicated for bilateral maxillary first premolar extraction, Subjects who have completed the alignment and levelling stage(19*25'SS), No systemic disease, No radiographic evidence of bone loss and No history of periodontal disease and habits such as smoking. Exclusion Criteria were; Previous history of orthodontic treatment, Class II division I malocclusion with extreme skeletal class II malocclusion, Severe vertical growers, Long term use of medication affecting tooth movement, Poor periodontal status and History of Smoking. A total of sixteen patients who fulfilled the inclusion criteria were selected for this split-mouth study. The two sides of each patient's maxillary arch were randomly allocated to the experimental and control groups using the coin-flip method. Following extraction of the maxillary first premolars, initial alignment & levelling was done. Following alignment and levelling phase, alginate impressions of the maxillary arch were obtained and poured immediately. The resulting dental casts were appropriately labelled for identification and later measurement.

The micro-osteoperforation procedure was carried out on the experimental side of maxillary arch. Patients were asked to rinse with 0.2% chlorhexidine and local anesthesia (2% lidocaine with 1:100,000 epinephrine) was infiltrated. The perforation sites were marked at the interdental depressions and soft-tissue thickness at these points was assessed with UNC probe. To standardize the penetration depth, a rubber stopper was placed on the mini-screw implant. Three perforations, each measuring 1.5 mm in diameter and 3 mm deep, were created in the buccal alveolar bone between the canine and second premolar using an implant driver. The first perforation was positioned 5 mm apical to free gingival margin, with subsequent perforations spaced 3 mm apart. Immediate canine retraction was started bilaterally using NiTi closed coil springs connected from molar hook to the canine bracket hook, delivering a calibrated 150-gram force. Anchorage was reinforced using transpalatal arch. Patients were scheduled at 4, 8 and 12-week intervals for reactivation of springs to maintain the 150-gram force bilaterally. At every visit, alginate impressions were taken, models poured and labelled for subsequent measurement.

## Determination of rate of canine retraction:

Vertical reference lines were marked on the palatal surfaces of both lateral incisor and canine, extending from the midpoint of incisal edge to the midpoint of cervical margin. The amount of canine movement was assessed by measuring the distance between the midpoints of middle third of lateral incisor and canine on both sides. These measurements were recorded before initiating canine retraction and again at 4, 8, and 12 weeks after retraction began. All assessments were carried out using digital vernier caliper with 0.1 mm precision.

## Determination of anchor loss:

To differentiate right and left sides, a 0.019 x 0.025' stainless steel L-shaped wire was inserted into the molar buccal tubes. The vertical arm of wire pointed gingivally on right side and occlusally on left side. Pre- and post-retraction lateral cephalograms were taken. The sella-nasion (SN) plane was traced and perpendicular line was drawn from sella. The perpendicular distance from this reference line to the terminal point of L-shaped molar wire was measured on both sides before & 12 weeks after start of canine retraction. The difference between pre- and post-retraction measurements indicated the amount of anchorage loss.

## Assessment of pain and discomfort:

Pain and discomfort were assessed using Visual Analogue Scale (VAS) scored from 1 to 10. Patients were instructed to record their level of discomfort immediately following MOP procedure, again after 24 hours and at the 4-week review appointment. SPSS statistical software version 23.0 was used to statistically analyze the collected data at P<0.05.

## Results:

The measurements obtained from experimental and control sides were compared and following results were recorded. In experimental group, the amount of canine retraction from baseline to 4 weeks was 0.84±0.126, from 4 weeks to 8 weeks was 0.52±0.092 and from 8 weeks to 12 weeks was 0.54±0.089. In control group, the amount of canine retraction from baseline to 4 weeks was 0.43±0.107, from 4 weeks to 8 weeks was 0.49±0.102 and from 8 weeks to 12 weeks was 0.51±0.091. There was a statistically considerable variation in mean amount of canine retraction between 2 groups at the end of 4 weeks with 5% significance level (i.e. <0.05). But it was not statistically significant from 4-8 weeks and 8-12 weeks (Table 1 - see PDF). In experimental group, the distance from line (perpendicular to SN plane) to vertical segment of L-shaped wire (terminal point) pre-canine retraction was 27.96±3.83 and at the end of 12 weeks was 28.72±3.79. In control group, the distance from line (perpendicular to SN plane) to vertical segment of L-shaped wire (terminal point) before start of canine retraction was 26.90±4.45 and at the end of 12 weeks was 27.75±4.50. The current study has shown that the amount of anchor loss at the end of 12 weeks was 0.77±0.345 in experimental group and 0.86±0.137 in control group. There was a statistically insignificant difference in mean amount of anchor loss between 2 groups at the end of 12 weeks (Table 2 - see PDF). The pain level immediately after the procedure was 1.56±1.094 in experimental group and 0.88±0.806 in control group and the difference was statistically insignificant. No pain and discomfort was reported after 24 hours and 4 weeks post MOP procedure in both groups (Table 3 - see PDF).

## Discussion:

Orthodontics has achieved remarkable progress in terms of precision, biomechanics and patient care. However, time continues to be the most unconquered frontier in pursuit of treatment acceleration. The prolonged treatment duration can lead to patient burnout and several adverse effects [2]. Consequently, it leads to the development of treatment modalities that can effectively reduce treatment duration without compromising treatment quality. Both surgical and non-surgical adjuncts have been proposed to enhance OTM rate. Among these, the surgical interventions have yielded favourable outcomes and enhanced long term stability in human clinical trials. However, the invasiveness, postoperative discomfort and the associated financial burden, often limits their acceptability and routine use in orthodontic practice. Among the surgical adjuncts, micro-osteoperforation and piezocision procedures cause minimal discomfort and are relatively conservative [1, 6]. However, piezocision requires specialized equipment and involvement of other specialists, limiting its practicality in a routine orthodontic setting. Micro-osteoperforation offers a simpler and more accessible alternative. It can be performed by an orthodontist using routinely available mini-implants. Micro-osteoperforations have been shown to stimulate the release of naturally occurring inflammatory mediators thereby promoting OTM [5]. However, despite the biological plausibility and encouraging preliminary reports, various studies have produced differing results regarding the efficiency of MOP in accelerating tooth movement. Hence, this study was designed to evaluate the effect of micro-osteoperforations on the rate of OTM. A split-mouth study design was chosen to minimize individual variability in biological response, tissue density and bone metabolism [7]. The study included both genders as human studies have generally found no significant sex differences in tooth movement rate [8]. The study also included biologically active age group (16-30 years) as bone turnover and osteoclastic activity usually decrease with age, potentially reducing OTM rate [9]. Randomization was done to avoid any bias related to dominant chewing side. Maxillary canine was chosen as it presents minimal occlusal interference in Class II Division 1 cases and typically undergoes greater and faster movement under equivalent orthodontic forces [10]. Treatment was started using Pre-adjusted Edgewise Appliance (MBT Prescription, 0.022' slot). After extraction and alignment stages, patients were prepared for MOP procedure. To achieve a catabolic effect of MOP, 3-4 perforations with penetration depths of 3-7 mm into the bone are recommended [5]. So, three MOPs of 3 mm depth were performed on the experimental side using mini-implants (1.5x6 mm). Maxillary canine retraction was initiated bilaterally using NiTi closed-coil springs delivering a standardized optimal force of 150 g per side [11]. The applied force was re-caliberated to 150 g at each visit to maintain standardization. The data indicate that MOP produced a transient but significant increase in tooth movement during the initial four weeks post-procedure. However, this effect diminished after the first month, with subsequent rates of tooth movement resembling those of the control side. This decline aligns with the expected biological timeline, as heightened cytokine concentration and osteoclastic presence following MOP gradually decline after four weeks [5]. These findings were consistent with those reported by Alikhani *et al.* who demonstrated 2.3-fold increase in canine retraction rate during the first 28 days following MOP [1]. The results also correlate with the systematic review of Shahabee [12] and studies by Parihar [13] and Feizbakhsh [14]. All those reported comparable findings-an initial acceleration followed by normalization of movement rate in subsequent months. Micro-ostoperforation only accelerated tooth movement over the first four weeks, according to Raghav *et al.* [15]. However, Jiaojiao [16] and Alkebsi [8] reported non-significant results over 12-weeks period. Such discrepancies may be attributed to variations in MOP protocol as well as differences in applied orthodontic forces and patient-specific biological variability. Anchorage loss was minimal and statistically similar between groups over a period of 12 weeks. These results correspond closely with those reported by Bokas and Wood [17]. By weakening the cortical bone around the canine and preserving the integrity of bone in the molar region, surgical procedures decrease the anchorage loss in the molar region [2]. The present findings align with studies by Alkebsi [8] and Aboalnaga [18] suggesting that the technique does not compromise anchorage control. Pain levels measured using VAS was low and comparable between groups. The mild increase in discomfort was transient and resolved within 24 hours, with no complaints thereafter. These results support the notion that MOP is well-tolerated and induces only minimal inflammatory discomfort. It was corroborated by previous studies of Alikhani [1], Alkebsi [8], Parihar [13] & Jiaojiao [16]. Alam *et al.* and Sugimori *et al.* concluded that, Micro-osteoperforations can efficiently accelerate orthodontic tooth movement in orthodontic patients [19, 20].

## Advancement to knowledge:

By inducing a regional accelerated phenomenon (RAP), micro-osteoperforations (MOPs) function as a minimally invasive, flapless surgical technique that speeds up orthodontic tooth movement (OTM). Teeth can move more quickly thanks to this method, which produces controlled micro-trauma in the alveolar bone that speed up bone remodeling and decrease the density of the surrounding cortical bone.

## Conclusion:

We show that micro-osteoperforations can effectively accelerate the rate of orthodontic tooth movement. The acceleration appears transient, lasting for approximately four weeks, after which the rate of movement parallels that of conventional mechanics. Importantly, the procedure does not increase tipping, anchorage loss or patient discomfort. Thus, MOP represents a biologically sound and clinically feasible adjunct for accelerating tooth movement in orthodontic practice.
